# *SOX5*-Null Heterozygous Mutation in a Family with Adult-Onset Hyperkinesia and Behavioral Abnormalities

**DOI:** 10.1155/2017/2721615

**Published:** 2017-10-29

**Authors:** Michael Zech, Katharina Poustka, Sylvia Boesch, Riccardo Berutti, Tim M. Strom, Wolfgang Grisold, Werner Poewe, Juliane Winkelmann

**Affiliations:** ^1^Institut für Neurogenomik, Helmholtz Zentrum München, Munich, Germany; ^2^Klinik und Poliklinik für Neurologie, Klinikum rechts der Isar, Technische Universität München, Munich, Germany; ^3^Kaiser-Franz-Josef-Spital Wien, Vienna, Austria; ^4^Department of Neurology, Medical University Innsbruck, Innsbruck, Austria; ^5^Institut für Humangenetik, Helmholtz Zentrum München, Munich, Germany; ^6^Institut für Humangenetik, Technische Universität München, Munich, Germany; ^7^Munich Cluster for Systems Neurology, SyNergy, Munich, Germany

## Abstract

*SOX5* encodes a conserved transcription factor implicated in cell-fate decisions of the neural lineage.* SOX5* haploinsufficiency induced by larger genomic deletions has been linked to a recognizable pediatric syndrome combining developmental delay with intellectual disability, mild dysmorphism, inadequate behavior, and variable additional features including motor disturbances. In contrast to* SOX5*-involving deletions, examples of pathogenic* SOX5* small coding variations are sparse in the literature and have been described only in singular cases with phenotypic abnormalities akin to those seen in the* SOX5* microdeletion syndrome. Here a novel* SOX5* loss-of-function point mutation, c.13C>T (p.Arg5X), is reported, identified in the course of exome sequencing applied to the diagnosis of an unexplained adult-onset motor disorder. Aged 43 years, our female index patient demonstrated abrupt onset of mixed generalized hyperkinesia, with dystonic and choreiform movements being the most salient features. The movement disorder was accompanied by behavioral problems such as anxiety and mood instability. The mutation was found to be inherited to the patient's son who manifested abnormal behavior including diminished social functioning, paranoid ideation, and anxiety since adolescence. Our results expand the compendium of* SOX5* damaging single-nucleotide variation mutations and suggest that S*OX5* haploinsufficiency might not be restrictively associated with childhood-onset syndromic disease.

## 1. Introduction

*SOX5*, which codes for the transcription factor SOX-5 on chromosome 12p12.1, is a pivotal regulator of gene-expression profiles during neurogenesis and thus essential in preserving nervous system integrity [1]. A role for* SOX5* haploinsufficiency in the manifestation of genetic disease in humans was first appreciated by Lamb and colleagues in a 2012 publication [2], describing monoallelic perturbation of* SOX5* in 16 individuals with a neurodevelopmental disorder characterized by intellectual disability, speech delay, dysmorphia, behavior problems, and variable other symptoms including motor dysfunction (referred to as Lamb-Shaffer syndrome, MIM616803). In these original patients, haploinsufficiency of* SOX5* was induced by DNA structural variations, mostly microdeletions (*SOX5* partial or full gene deletions), in the 12p12.1 chromosomal region, as demonstrated by array comparative genomic hybridization diagnostics. Several subsequent studies reporting on the identification of independent patients with deletions encompassing* SOX5* confirmed these initial findings and contributed to the definition of* SOX5*-related disease as a multisystemic pediatric syndrome [3–5]. More recently, the first intragenic* SOX5* point mutation, a unique pathogenic stop-gain alteration, was detected by whole-exome sequencing (WES) in a patient featuring relevant phenotypic overlap with the* SOX5* microdeletion patients [6]. Finally, in the search for novel molecular factors underlying intellectual disability, a 2016 large-scale trio-based whole-exome study discovered significantly more* SOX5* loss-of-function coding mutations than expected among ~2,100 affected individuals [7]. These patients were reported to have intellectual disability with additional features of language delay, facial dysmorphic signs, and behavior deficits, consistent with the previously delineated* SOX5* haploinsufficiency phenotype. Although the genetic and clinical determinants of this newly identified syndrome have begun to be elucidated, our understanding of the* SOX5* mutational landscape as well as the entire phenotype spectrum linked to* SOX5* haploinsufficiency is incomplete and awaits further investigation. In this report, we extend the role of heterozygous* SOX5* deficiency in human disease by describing an Austrian family with an index patient primarily enrolled in a study of the genetic aetiology of an adult-onset movement disorder.

## 2. Case Presentations

**Figure 1:**
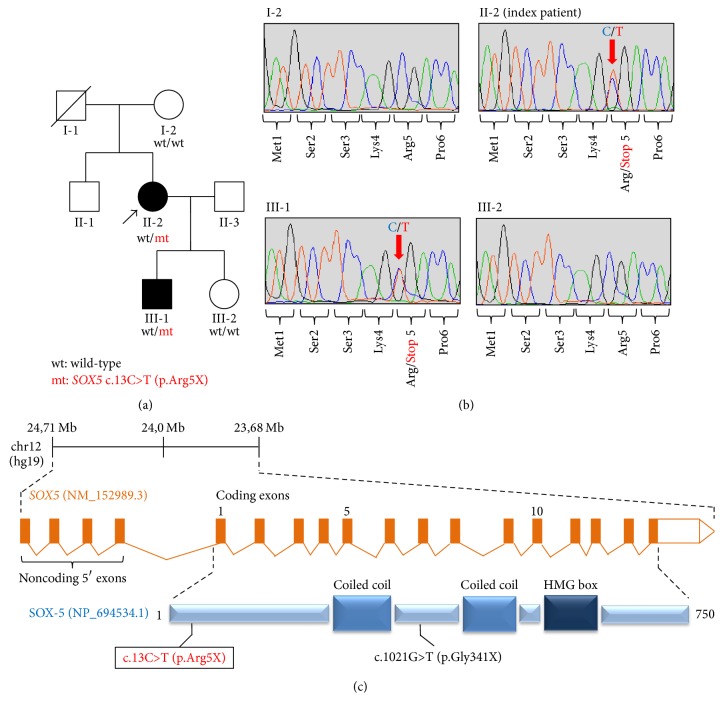
Loss-of-function short coding mutations in* SOX5*. (a) Pedigree of the family investigated in this study.* SOX5* mutational status is shown below each tested family member. Symbols are as follows: circles, females; squares, males; filled, phenotypically abnormal; empty, healthy; slash, deceased. (b) Dideoxy sequencing traces document* SOX5* c.13C>T (p.Arg5X) in genomic DNA of the index patient II-2 and her son III-1. Individuals I-2 and III-2 show homozygous wild-type sequence at this site. Arrows indicate the mutant nucleotide positions. (c) Schematic overview of the human* SOX5* locus (NM_152989.3; top) and its encoded protein (NP_694534.1; bottom) illustrates the newly identified c.13C>T (p.Arg5X) stop-gain variant (boxed) and a loss-of-function point mutation previously described [6]. Notably, all of the critical SOX-5 protein domains are distal to the p.Arg5X protein-truncation site and thus the truncated protein, if expressed, is highly unlikely to retain residual function. The schematic is simplified and not drawn to exact scale.

The index patient, individual II-2 in Figure 1(a), was a 47-year-old woman of Austrian descent. She had an older, healthy brother (individual II-1). Family history was unremarkable on both the maternal and paternal side. The index patient first developed gait clumsiness and adventitious movements of the trunk and arms while walking at the age of 43 years. These symptoms were relatively sudden in onset (days) but the patient was unaware of any triggering factor. Over the next few months, the patient progressively manifested continuous abnormal involuntary movements affecting her face, neck, trunk, and all 4 extremities. Concurrently, she noted intermittent worsening of lower-limb incoordination. She reported impairment of fine motor tasks, with dropping of objects when under psychological stress. At this time, she consulted several local medical services and neurologists who interpreted her condition as “idiopathic choreo-dystonic syndrome” or “generalized dyskinesia of unknown origin.” Given her acute presentation of motor impairment, a functional movement disorder was also discussed but deemed unlikely because of the lack of additional signs and symptoms characteristic of a psychogenic illness [8]. Extensive routine diagnostic work-up including systematic blood chemical analysis, screening for acanthocytes, laboratory testing for Wilson's disease, panel testing for autoimmune-encephalitis antibodies, antibody screening for neurotropic viruses, CSF analysis, electroencephalography, and brain MRI was unrevealing. Genetic testing for Huntington's disease was normal. Levodopa administration was unbeneficial. Aged 45 years, the patient was referred for reevaluation of her movement disorder to our department. The neurological examination revealed cervical dystonia, truncal dystonia, dystonic finger posturing, and severe orofacial dyskinesia. There were diffuse generalized choreiform movements, more pronounced in her shoulders and arms compared to lower limbs. She displayed unsteady gait with trunk instability secondary to irregular unpredictable movements but she could walk unaided. The remainder of the examination was unremarkable. No cerebellar pathological signs, pyramidal tract abnormalities, sensory involvement, or autonomic involvement were observed. Visual acuity and hearing were normal, as were extraocular movements, deep tendon reflexes, and flexor-plantar-responses. Moreover, no systemic pathological features were evident. The patient's history was uninformative in terms of delivery and development of motor and intellectual milestones. She had attended a regular school with only minor learning disability. Her history was, however, significant for an episode of anxiety and mood swings. Between the ages of 30 and 35 years, she was treated with selective serotonin reuptake inhibitors to manage her mood disorder, which was controlled since then. Notably, there was no medication regimen with neuroleptics or any other antidopaminergic agents prior to the appearance of her hyperkinesia and therefore a pharmacological effect explaining the movement disorder cannot be suspected. At present, more than 4 years into her motor disease, the patient continued to have persistent mixed generalized hyperkinesia with pronounced dystonic and choreic elements. Symptoms were unremitting, while the patient was started on tetrabenazine with some subjective improvements in motor capabilities.

The patient had 2 children, an asymptomatic daughter (individual III-2) and an older, phenotypically abnormal son (individual III-1). The son was a 27-year-old man who was born at term after an uncomplicated pregnancy. His developmental milestones were achieved normally. Although there was no evidence of cognitive decline, he required extratutoring at school and was described as showing oppositional reactions and aggression toward his peers. He first came to medical attention at the age of 20 years due to the presentation of paranoid beliefs and difficulties in executive functioning. According to her mother, he also experienced significant anxiety. During his visit at our department at the age of 26 years, physical examination was performed without any substantial findings. In particular, there were no peculiar facial characteristics or abnormal hyperkinetic movements.

## 3. Materials and Methods

To explore the genetic underpinnings of disease in the Austrian family, we collected peripheral blood samples of the index patient II-2, her mother I-2, her son III-1, and her daughter III-2 after obtaining informed consent under institutional-review-board-approved protocols. Genomic DNA was extracted using standard procedures. We conducted WES from DNA of the index patient by using the SureSelect Human All Exon 50 Mb Kit (v.5, Agilent Technologies) for the generation of capture libraries. Massively parallel sequencing was done on a HiSeq2500 next-generation sequencing instrument (Illumina) in paired-end (100-bp) mode with a median read depth of 185-fold. Data were aligned to a modified human reference genome (hg19/GRCh37) and high-confidence variant calls were produced as previously described [9]. Subsequently, nonsilent variants (coding excepting nonsplicing synonymous and splicing mutations) were retained when their minor allele frequency was <0.001 in ~60,000 exomes from the Exome Aggregation Consortium (ExAC) Browser and an in-house dataset of ~10,000 ethnically matched exome-sequenced individuals. Next, filtered variants were considered to be disease-related if they resided in genes previously associated with key clinical symptoms observed in the index patient. To this end, we interrogated candidate gene lists obtained from the scientific literature [9]. These lists were curated manually and created via exhaustive phenotype-based searches (analytical phenotype search keywords: “dystonia”; “chorea”; “hyperkinesia”; “dyskinesia”; “clumsiness”) in original publications provided by PubMed (https://www.ncbi.nlm.nih.gov/pubmed/) and website entries provided by the Online Mendelian Inheritance in Man (OMIM) database (https://www.omim.org/).

Dideoxy sequencing was performed to confirm the variant selected as potentially relevant to disease and test for genotype-phenotype cosegregation in the family. Amplification primers were designed with ExonPrimer (https://ihg.helmholtz-muenchen.de/ihg/ExonPrimer.html).

## 4. Results

Phenotypically guided filtering of the index patient's WES data left a single heterozygous, predicted detrimental single-nucleotide variant in* SOX5*, a gene previously linked to the phenotypes hyperkinesia and clumsiness. At nucleotide position 13 of the* SOX5* brain-expressed long transcript (NM_152989.3; coding exon 1; Figure 1(c)), we identified a C-to-T substitution leading to the insertion of a premature stop codon at amino acid 5 (p.Arg5X). As a result, this variation was expected to either trigger nonsense-mediated messenger-RNA decay or give rise to a truncated SOX-5 that is highly unlikely to retain functionality (Figure 1(c)).* SOX5* c.13C>T (p.Arg5X) was therefore presumed to be a null allele, resulting in the inactivation of one* SOX5* copy in the index patient. The variant was absent from ~60,000 population controls in ExAC and ~10,000 internal control individuals sequenced by exome. It was referenced in dbSNP142 (rs780962501) with 1 occurrence in the Genome Aggregation Database (gnomAD). The mutation was verified through dideoxy sequencing in the index patient using primers flanking the mutated base and shown to be present in her phenotypically abnormal son (III-1) (Figure 1(b)). The index patient's mother (I-2) and daughter (III-2) were each found to be homozygous for the reference allele.

## 5. Discussion

*SOX5* encodes a member of the SRY-HMG-box- (SOX-) containing family of evolutionary conserved transcription factors involved in fundamental developmental pathways across multiple tissues [10]. SOX-family genes are subdivided into 10 categories (A–J), in which* SOX5*, along with* SOX6* and* SOX13*, belongs to the SOXD group [10]. Expression of the long* SOX5* transcript (NM_152989.3) was found to be abundant in human and mouse brain from early embryonic stages [11]. Significant amounts of past work have gone into the role of* SOX5* in the maturation of the central nervous system, demonstrating that the gene is vital to the modulation of critical fate decisions, proliferation, and differentiation of various neural progenitors [1, 12, 13]. Mice with two* SOX5* null alleles die in the early postnatal period [14] and their brains show severe defects in the generation of neuronal subcortical projections [12]. Similarly, a recent transgenic* Drosophila* model of* SOX5* underscored its critical role in giving rise to proper neuronal network formation [15]. Considering the developmental significance of* SOX5*, it is not surprising that* SOX5* dosage-reduction in humans is not tolerated and that heterozygous loss-of-function mutations in* SOX5* are relevant to human disease. These suppositions are also well reflected in statistical measures gained by large population-based variation resources, such as ExAC, indicating that* SOX5* is under intense selective constraint for loss-of-function alterations (probability of being loss-of-function intolerant [pLI] score 1.0) [16]. In prior literature, multiple genomic structural variations disrupting* SOX5* have been described in patients but only a highly restricted number of pathogenic short intragenic mutations have been recognized. By means of WES, we detected here an additional* SOX5* stop-gain variant (c.13C>T [p.Arg5X]), raising to two the number of detailed clinical reports on individuals with deleterious* SOX5* coding mutations. The fact that the c.13C>T (p.Arg5X) allele is annotated in dbSNP142 and found once in the gnomAD Browser does not conflict with the hypothesis of a clinically relevant mutation given that (1) the dbSNP database is known to contain rare pathogenic variants and (2) gnomAD includes sequence information from phenotypically abnormal individuals [16, 17]. In particular, gnomAD incorporates data of trio sequencing performed on families with neuropsychiatric disease, a central phenotypic aspect observed in the current patients. Although* SOX5* c.13C>T (p.Arg5X) was identified in the index patient's WES dataset via a clinically driven filtering strategy using the phenotype search keywords “hyperkinesias” and “clumsiness”—2 symptoms previously correlated with heterozygous* SOX5* deficiency—the clinical courses observed in our mother-son pair differed from those previously reported in association with* SOX5* haploinsufficiency. The index patient was ascertained in the adult neurology setting with a delayed-onset movement disorder, whereas the previously described patients manifested a constellation of deficits characteristic of a pediatric neurodevelopmental syndrome [2]. Likewise, the index patient's son showed no evidence of developmental or intellectual regression but presented with severe behavioral abnormalities throughout adolescence. Thus, our results indicate that the* SOX5*-related phenotype extends well beyond childhood-onset syndromic disease. Of note,* SOX5* has also been demonstrated to play an important role during chondrogenesis [10], but there were no skeletal deformities or morphological stigmata compatible with cartilage defects in the* SOX5* disease subjects described here.

We note that we do not here provide unambiguous evidence to attribute the patients'* SOX5* stop-gain mutations to their phenotypes, but nevertheless there are strong arguments in favor of a causal relationship: (i) from a genetics viewpoint, transcripts generated by* SOX5* c.13C>T (p.Arg5X), regardless of whether they are subjected to nonsense-mediated decay or evade this mechanism, are highly likely to be nonfunctional (Figure 1(c)). Thus, c.13C>T (p.Arg5X) is a predicted null allele in a haploinsufficient gene. Furthermore, c.13C>T (p.Arg5X) was exclusive to phenotypically abnormal individuals in comparison to the rest of the family and we were unable to identify any other variant in the index patient's WES data that accounted for her phenotype; (ii) from a phenotypic viewpoint, the index patient and her son shared some core clinical features with previously described* SOX5*-haploinsufficient patients [2]. In fact, hyperkinesia and other types of motor dysfunction were recurrent, albeit variable findings among described* SOX5*-mutated subjects, whereas behavior deficits—as evident in both the mother and her son—were almost universally present. Accordingly, the observation of a more restricted disease phenotype in the patients reported here suggests that family-specific presentations outside the more commonly seen phenotypic domains may exist in* SOX5*-related disease. Variable expressivity has been previously recognized between and within* SOX5*-mutated families and it is a very well-known phenomenon in a multitude of neurodevelopmental disorders. In this context, it is also worth mentioning that recent work has found an enrichment of* SOX5* missense mutations in patients with age-related neurodegeneration, hinting at an even broader role for* SOX5* in human disease phenotypes [15]. Although it remains to be determined why individual patients are differentially affected by* SOX5* mutations, it is possible that the ultimate phenotypic outcome depends on the interaction of their biological effects with modifier genes, genetic background, and environmental exposures; (iii) from a mechanistic viewpoint,* SOX5* haploinsufficiency appears to be a plausible pathophysiological event underlying the observed phenotypes. Hyperkinetic movement disorders and behavior deficits are genetically profoundly heterogeneous conditions that arise as a result of mutations in genes involved in various biologically relevant activities. For both pathologies, however, heterozygous disruption of transcription factor-encoding genes has emerged as an important disease mechanism [18, 19]. Furthermore, it is especially interesting to consider that a connection between movement disorders, in particular dystonia, and mutations of other SOX-family genes has been proposed previously by separate studies. Ebrahimi-Fakhari and colleagues reported a patient with generalized dystonia who was found to harbor a de novo heterozygous deletion in* SOX6*, another member of the SOXD subgroup [20]. Remarkably, reminiscent of the presentation seen in our* SOX5*-mutated index patient, dystonia in the* SOX6* deletion patient was of acute onset and combined with other hyperkinesia. In addition, Bakrania and colleagues described lower-limb dystonia in association with a heterozygous protein-truncating mutation in* SOX2* [21].

To conclude, we have identified an ultrarare, previously undescribed* SOX5* loss-of-function allele in a family with adult-onset abnormal hyperkinetic movements and psychiatric disturbances, thereby suggesting an expansion of the genotypic and clinical spectrum linked to* SOX5* haploinsufficiency. Future studies are warranted to further explore a possible role of* SOX5* in movement and neuropsychiatric disorders and advance knowledge of the clinical heterogeneity that arises upon inactivation of one* SOX5* copy in humans. Given the phenotypic variability that is indicated by the current investigation, we posit that the presence of hyperkinesia in both the context of a childhood neurodevelopmental disorder and a late-onset acute presentation should prompt a search for heterozygous* SOX5* mutations in array diagnostics, exome sequencing, or genome-sequencing data. The breath of phenotypic variation related to mutations in* SOX5* is likely to increase when additional patients will be identified over the coming years.
